# A Ceftazidime-Avibactam-Resistant and Carbapenem-Susceptible Klebsiella pneumoniae Strain Harboring *bla*_KPC-14_ Isolated in New York City

**DOI:** 10.1128/mSphere.00775-20

**Published:** 2020-08-26

**Authors:** Siqiang Niu, Kalyan D. Chavda, Jie Wei, Chunhong Zou, Steven H. Marshall, Puneet Dhawan, Deqiang Wang, Robert A. Bonomo, Barry N. Kreiswirth, Liang Chen

**Affiliations:** a Department of Laboratory Medicine, The First Affiliated Hospital of Chongqing Medical University, Chongqing, China; b Hackensack Meridian Health Center for Discovery and Innovation, Nutley, New Jersey, USA; c Department of Laboratory Medicine, West China Second University Hospital, and Key Laboratory of Obstetric & Gynecologic and Pediatric Diseases and Birth Defects of Ministry of Education, Sichuan University, Chengdu, China; d College of Laboratory Medicine, Chongqing Medical University, Yuzhong District, Chongqing, China; e Research Service, Louis Stokes Cleveland VA Medical Center, Cleveland, Ohio, USA; f Molecular Resource Facility and Department of Microbiology, Biochemistry and Molecular Genetics, New Jersey Medical School, Rutgers University, Newark, New Jersey, USA; g The Key Laboratory of Molecular Biology of Infectious Diseases designated by the Chinese Ministry of Education, Chongqing Medical University, Yuzhong District, Chongqing, China; h Departments of Medicine, Pharmacology, Molecular Biology and Microbiology, Case Western Reserve University, Cleveland, Ohio, USA; i Case VA Center for Antimicrobial Resistance and Epidemiology (CARES), Cleveland, Ohio, USA; j Department of Medical Sciences, Hackensack Meridian School of Medicine, Nutley, New Jersey, USA; JMI Laboratories

**Keywords:** ceftazidime-avibactam, carbapenem, KPC, plasmid, antimicrobial resistance, selection

## Abstract

Ceftazidime-avibactam is a potent antibiotic combination against Klebsiella pneumoniae carbapenemase (KPC)-producing *Enterobacteriaceae*. Here, we describe a unique ceftazidime-avibactam-resistant and carbapenem-susceptible K. pneumoniae strain harboring a novel *bla*_KPC-14_ variant. This strain was isolated from a New York City patient in 2003, which predates the introduction of avibactam. Despite resistance to ceftazidime-avibactam, the strain was susceptible to imipenem-relebactam and meropenem-vaborbactam. Comprehensive genomic sequencing revealed that *bla*_KPC-14_ is harbored on an ST6 IncN plasmid associated with the early spread of *bla*_KPC_.

**IMPORTANCE** KPC is currently the most common carbapenemase identified in the United States. More than 40 KPC variants have been described, of which KPC-2 and KPC-3 are the most frequent clinical variants. However, our understanding of the genetic structures and β-lactam resistance profiles of other novel KPC variants remains incomplete. Here, we report a novel *bla*_KPC_ variant (*bla*_KPC-14_) and the complete genome sequence of *bla*_KPC-14_-harboring K. pneumoniae strain BK13048, which is susceptible to carbapenems but resistant to ceftazidime-avibactam. To the best of our knowledge, this is one of the earliest KPC-producing K. pneumoniae strains exhibiting resistance to ceftazidime-avibactam.

## Abstract (precis)

KPC is currently the most common carbapenemase identified in the United States. More than 40 KPC variants have been described, of which KPC-2 and KPC-3 are the most frequent clinical variants. However, our understanding of the genetic structures and β-lactam resistance profiles of other novel KPC variants remains incomplete. Here, we report a novel *bla*_KPC_ variant (*bla*_KPC-14_) and the complete genome sequence of *bla*_KPC-14_-harboring K. pneumoniae strain BK13048, which is susceptible to carbapenems but resistant to ceftazidime-avibactam. To the best of our knowledge, this is one of the earliest KPC-producing K. pneumoniae strains exhibiting resistance to ceftazidime-avibactam.

## OBSERVATION

The rapid spread of carbapenemases among members of the *Enterobacteriaceae* family poses a major clinical concern, since it greatly limits therapeutic options. These β-lactamases are capable of hydrolyzing all generations of cephalosporins and carbapenems, the last-resort antibiotics for complicated infections with multidrug-resistant Gram negative bacteria. Among the carbapenemases, Klebsiella pneumoniae carbapenemase (KPC), an Ambler class A serine β-lactamase, is particularly problematic, with major outbreaks in the northeastern United States, followed by its spread throughout the United States and worldwide (1). As a novel combination of a β-lactam and a β-lactamase inhibitor, ceftazidime-avibactam was highly active against KPC-producing bacteria. However, resistance to ceftazidime-avibactam has also been reported in patients who were treated with this combination, primarily due to amino acid substitutions in the KPC β-lactamase (2–4). In this study, we describe a novel KPC variant, KPC-14, isolated from K. pneumoniae strain BK13048, collected in 2003 from a New York City (NYC) patient. Surprisingly, this strain was both susceptible to carbapenems and resistant to ceftazidime-avibactam, a result indicating that this resistant KPC variant existed prior to 2015, when ceftazidime-avibactam was introduced.

Strain BK13048 was identified as a part of retrospective study screening of extended-spectrum cephalosporins and carbapenem-resistant K. pneumoniae from our archived strain collection. A molecular-beacon-based allelic discrimination real-time PCR assay (5) showed that strain BK13048 harbored a *bla*_KPC-6-like_ variant. PCR and Sanger sequencing of the full-length *bla*_KPC_ gene revealed a novel *bla*_KPC_ variant, *bla*_KPC-14_. Nucleotide alignment of different *bla*_KPC_ variants showed that *bla*_KPC-14_ differs from *bla*_KPC-2_ by a 6-bp deletion (nucleotide positions 721 to 726), resulting in a 2-amino-acid deletion at Ambler positions 242Gly and 243Thr. KPC-28 has the same 242Gly and 243Thr deletion, but an additional His274Tyr substitution distinguishes this variant from KPC-14 (6).

Broth microdilution susceptibility testing showed that BK13048 is resistant to ceftriaxone (MIC, >16 μg/ml), ceftazidime (MIC, >256 μg/ml), piperacillin (MIC, >1,024 μg/ml), aztreonam (MIC, >64 μg/ml), and ceftazidime-avibactam (MICs, >16 and 4 μg/ml) but susceptible to imipenem (MIC, ≤0.25 μg/ml), ertapenem (MIC, ≤0.03 μg/ml), and meropenem (MIC, ≤0.03 μg/ml).

The MIC results from BK13048 showed an unusual profile: susceptibility to carbapenems but resistance to ceftazidime-avibactam. To investigate this finding, the full-length *bla*_KPC-2_, *bla*_KPC-3_, and *bla*_KPC-14_ genes and the same promoter sequences were cloned into pET28a vectors, followed by electroporation into Escherichia coli DH10B cells (Invitrogen). Susceptibility testing of the *bla*_KPC-14_, *bla*_KPC-2_, and *bla*_KPC-3_ E. coli DH10B constructs showed that the *bla*_KPC-14_ construct demonstrated a ceftazidime-avibactam MIC of >16 μg/ml, which is at least 64-fold higher than that of the *bla*_KPC-2_ or *bla*_KPC-3_ construct (MICs, ≤0.25 μg/ml) (Table 1). Similarly, the MIC of ceftazidime was much higher for the *bla*_KPC-14_ plasmid construct (256 μg/ml) than for the cloned *bla*_KPC-2_ (4 μg/ml) or *bla*_KPC-3_ (16 μg/ml) gene.

**TABLE 1:** Susceptibilities of the strains studied to β-lactams

Strain	Description	Carbapenemase	MIC^*a*^ (μg/ml)								
			PIP	TZP	CRO	CAZ	ETP	MEM	IMP	ATM	CAZ-AVI
BK13048	Clinical isolate	*bla*_KPC-14_	1,024	8	16	256	≤0.03	≤0.03	0.25	64	>16
KPC2-pET28	*bla*_KPC-2_-harboring E. coli DH10B	*bla*_KPC-2_	128	32	8	4	1	2	2	16	0.25
KPC3-pET28	*bla*_KPC-3_-harboring E. coli DH10B	*bla*_KPC-3_	128	32	8	16	1	1	2	32	0.25
KPC14-pET28	*bla*_KPC-14_-harboring E. coli DH10B	*bla*_KPC-14_	32	4	8	256	≤0.03	≤0.03	0.25	32	>16
E. coli DH10B			1	1		0.25	≤0.03	≤0.03	≤0.03	≤0.125	≤0.03

a PIP, piperacillin; TZP, piperacillin-tazobactam; CRO, ceftriaxone; CAZ, ceftazidime; ETP, ertapenem; MEM, meropenem; IMP, imipenem; ATM, aztreonam; CAZ-AVI, ceftazidime-avibactam.

In contrast, the MICs of the different carbapenems (ertapenem, meropenem, and imipenem) for the *bla*_KPC-14_ construct were 8- to 32-fold lower than the MICs for the *bla*_KPC-2_ or *bla*_KPC-3_ construct (Table 1). The susceptibility testing results presented above were consistent with a previous study by Oueslati et al. testing KPC-14 and KPC-28 using a different plasmid vector (pTOPO) (6). Those results demonstrated that the 242Gly and 243Thr amino acid deletions in KPC-14 decreased carbapenem activity but increased potency against ceftazidime and ceftazidime-avibactam (Table 1), and ceftazidime-avibactam resistance is likely due to increased activity against ceftazidime rather than reduced inhibition against avibactam.

We further characterized and compared the kinetic parameters of KPC-14 and KPC-2. In brief, the sequences without the signal peptide (from *bla*_KPC-14_ and *bla*_KPC-2_) were obtained by PCR amplification using primers NdeI-KPC-2-F(30–293) (5′-ACGCATATGGCGGAACCATTCGCTAAAC-3′) and Xhol-KPC-2-R-STOPdel (5′-TAACTCGAGCTGCCCGTTGACGCCCAAT-3′), followed by insertion into plasmid pET28a in E. coli DH10B (Invitrogen). The KPC enzymes were then purified, and the steady-state kinetic parameters were determined as described previously (6, 7). The results showed that KPC-14 has a higher catalytic efficiency of ceftazidime and cefepime, but a lower hydrolysis activity of imipenem, than KPC-2 (Table 2). In addition, no meropenem hydrolysis could be detected with purified KPC-14 under current conditions (measurement made over 5 min). The hydrolytic profile of KPC-14 was similar to that in the previous report from Oueslati et al. (6) and was consistent with the MIC observations presented above. Moreover, a previous experiment determining the 50% inhibitory concentrations (IC_50_) of β-lactamase inhibitors also suggested that the 2-amino-acid 242Gly and 243Thr deletion had no impact on the inhibition properties of avibactam (6).

**TABLE 2:** Steady-state kinetic parameters of purified KPC-2 and KPC-14 β-lactamases

β-Lactam	KPC-2			KPC-14^*a*^		
	*k*_cat_ (s^−1^)	*K_m_* (μM)	*k*_cat_*/K_m_* (μM^−1^·s^−1^)	*k*_cat_ (s^−1^)	*K_m_* (μM)	*k*_cat_*/K_m_* (μM^−1^·s^−1^)
Meropenem	8.078	16.263	0.497	ND^*b*^	ND	ND
Imipenem	28.797	98.350	0.293	19.490	548.805	0.036
Ceftazidime	3.274	590.717	0.006	24.600	73.860	0.333
Aztreonam	12.601	2398.451	0.005	2.875	192.335	0.015
Cefepime	4.748	310.480	0.015	7.588	70.406	0.108
Piperacillin	7.709	793.526	0.010	1.084	45.767	0.024
Cefazolin	65.877	110.746	0.595	27.126	287.930	0.094

a KPC-14 differs from KPC-2 by a 2-amino-acid 242Gly and 243Thr deletion.

b ND, not detectable due to a low initial rate of hydrolysis.

Additional testing of BK13048 and the *bla*_KPC-14_ plasmid construct against other novel β-lactam–β-lactamase combinations, i.e., imipenem-relebactam and meropenem-vaborbactam (by disk diffusion assay), showed that they were susceptible to imipenem-relebactam (inhibition zone diameter, >28 mm for both strains) and meropenem-vaborbactam (inhibition zone diameter, >30 mm).

To better understand the genetic structure associated with the *bla*_KPC-14_ gene, comprehensive whole-genome sequencing was performed using a combination of the Oxford Nanopore MinION and Illumina HiSeq platforms, followed by hybrid assembly using Unicycler (8). The complete sequencing of BK13048 showed that it contains a 5,213,293-bp chromosome with an average G+C content of 57.6% and harbors 5,311 predicted open reading frames. In addition, it contains seven plasmids ranging from 5 kbp to 82 kbp. *In silico* multilocus sequencing typing (MLST) revealed that BK13048 belongs to sequence type (ST) 16 (9), which has been reported to cause nosocomial infections worldwide and is associated with *bla*_NDM-1_-encoded carbapenemase and the presence of the extended-spectrum beta-lactamase (ESBL) gene *bla*_CTX-M-15_ (10). Analysis of acquired antimicrobial resistance (11) identified 14 antimicrobial resistance genes encoding resistance to β-lactams, aminoglycosides, fluoroquinolones, fosfomycin, sulfonamide, and trimethoprim (Table 3). In addition, *in silico* plasmid replicon typing (12) indicated that the seven plasmids belong to IncA/C, F, M, N, R, ColRNAI, and a novel incompatibility group (Table 3).

**TABLE 3:** Key features of the genome and plasmids harbored by BK13048

Characteristic	Chromosome	pBK13048_1	pBK13048_2	pBK13048_3	pBK13048_KPC	pBK13048_5	pBK13048_6	pBK13048_7
Size (bp)	5,213,293	82,240	61,331	51,887	50,635	44,850	28,729	5,251
% G+C	57.6	51.9	50.8	52.8	53	53.6	53.5	49.2
β-Lactamase(s)	*bla*_SHV-1_	*bla*_OXA-9_, *bla*_TEM-1A_			*bla*_KPC-14_			
Other resistance genes	*fosA*, *oqxB*, *oqxA*	*aadA1*, *strA*, *strB*, *aac(6′)-Ib*, *sul2*			*dfrA14*			
Plasmid incompatibility (Inc)		A/C	M	New	N	R	F	ColRNAI

**FIG 1:**
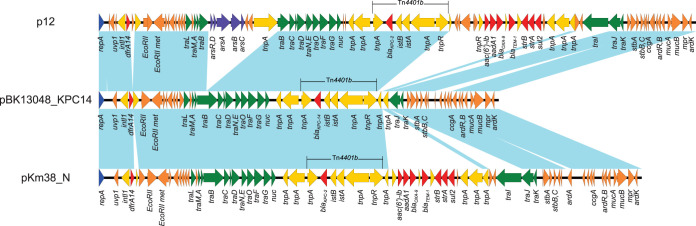
Structures of plasmids p12 (GenBank accession no. FJ223605), pBK13048_KPC14 (accession no. CP045022), and pKm38_N (accession no. KY128483). Colored arrows indicate open reading frames, with blue, orange, green, red, and purple arrows representing replication genes, plasmid backbone genes, mobile elements, plasmid transfer genes, and antimicrobial and heavy metal resistance genes, respectively. Blue shading indicates regions of shared homology among different elements.

The *bla*_KPC-14_ gene is located on an IncN plasmid, pBK13048_KPC14 (Table 1). pBK13048_KPC14 is 50,635 bp long with an average G+C content of 53% and carries *bla*_KPC-14_ on the Tn*4401b* transposon (Fig. 1). Full plasmid sequence BLAST against NCBI GenBank (http://blast.ncbi.nlm.nih.gov/Blast.cgi) showed that pBK13048_KPC14 is highly similar to plasmid pKm38_N from Klebsiella oxytoca, which was isolated in 1997 in New York City (13), with 100% query coverage and overall 99.98% sequence identity (Fig. 1). In addition, pBK13048_KPC14 showed 94% query coverage and overall 99.97% sequence identity to one of the first sequenced *bla*_KPC_-harboring IncN plasmids, plasmid 12, isolated from NYC in 2005 (Fig. 1) (14). In agreement with the structure of other IncN plasmids (13, 15), pBK13048_KPC14 contains a 2-kb acquired region integrated downstream of *uvp1* and harbors *dfrA14*, encoding trimethoprim resistance. In addition, pBK13048_KPC14 contains a second acquired region downstream of the *nuc* gene and carries *bla*_KPC-14_ (Fig. 1). This highlights the important role played by IncN plasmids in the spread of *bla*_KPC_ during the early years of the carbapenem resistance epidemic. *In silico* IncN plasmid MLST showed that pKm38_N (isolated in 1997), pBK13048_KPC14 (2003), and p12 (2005) all belong to ST6 (*repN*-*traJ*-*korA*, allele profile 2-4-2), which is different from the sequence type harboring the *bla*_KPC-28_-containing IncN plasmid pWI2-KPC28 (ST15, allele profile 7-6-3) from E. coli. Even though both KPC-14 and KPC-28 have the same 242Gly and 243Thr amino acid deletions, their genomic history suggests that pBK13048_KPC14 and pWI2-KPC28, as well as the *bla*_KPC-14_ and *bla*_KPC-28_ genes, likely evolved independently on different IncN plasmid backgrounds.

A recent study from Italy described the emergence of two ceftazidime-avibactam-resistant subpopulations of K. pneumoniae ST1685 (unrelated to the ST16 of BK14038), carrying KPC-14 and KPC-31 (Asp179Tyr substitution within the KPC Ω-loop), in a patient following prolonged ceftazidime-avibactam treatment (16). Our study also suggested that the ceftazidime-avibactam-resistant KPC variants, e.g., KPC-14, could exist even without ceftazidime-avibactam exposure. These KPC variants, with reduced carbapenem hydrolytic capacities, raise a challenge for phenotypic and genotypic carbapenemase detection tests, since some of these assays may classify KPC-14 strains as carbapenemase producers (6, 16). Consequently, molecular testing followed by a phenotypic carbapenemase activity assay has been proposed to detect and differentiate KPC variants associated with carbapenem susceptibility and ceftazidime-avibactam resistance (16).

Taken together, we identified, completely sequenced, and characterized a novel *bla*_KPC_ variant from K. pneumoniae BK13048, designated *bla*_KPC-14_, that revealed an unexpected resistance to ceftazidime-avibactam. Comprehensive sequence analysis and assembly using both the Illumina and Oxford Nanopore platforms revealed the genetic changes in *bla*_KPC-14_ and its plasmid structure. In contrast to other ceftazidime-avibactam-resistant *bla*_KPC_ variants, the *bla*_KPC-14_ gene was not under ceftazidime-avibactam selection pressure, as evidenced by the fact that the isolation of BK13048 predated the U.S. introduction of this novel β-lactam and β-lactamase inhibitor combination in 2015.

### Accession number(s).

The complete nucleotide sequence of strain BK13048 has been deposited in GenBank as accession no. CP045015 to CP045022.
